# Multidisciplinary treatment of patients with diabetes and hypertension: experience of a Brazilian center

**DOI:** 10.1186/s13098-017-0305-2

**Published:** 2018-01-08

**Authors:** Thiago Veiga Jardim, Sayuri Inuzuka, Luan Galvão, Leandra Anália Freitas Negretto, Rogério Orlow de Oliveira, Wanessa Faria Sá, Haroldo Silva de Souza, Andrea Crisitina Sousa, Patricia Silva Carneiro, Weimar Kunz Sebba Barroso, Ana Luiza Lima Sousa, Paulo César Veiga Jardim

**Affiliations:** 1 Hypertension League, Federal University of Goias, 1ª Avenida, S/N.-Setor Universitário, Goiânia, GO CEP 74085-300 Brazil; 2 Division of Cardiovascular Medicine, Brigham & Women’s Hospital, 75 Francis Street, Boston, MA 02115 USA; 3 Center for Health Decision Science, Harvard TH Chan School of Public Health-Department of Health Policy and Management, 718 Huntington Avenue, 2nd Floor, Boston, MA 02115 USA

**Keywords:** Hypertension, Diabetes, Multidisciplinary treatment

## Abstract

**Background:**

Although multidisciplinary treatment is recommended for type 2 diabetes mellitus and hypertension (HTN), there is a lack of scientific literature supporting the hypothesis of extending this treatment strategy to patients with both diabetes and HTN. Aiming to report results of long-term multidisciplinary treatment for these patients and identify strategies to improve their management, we conducted this study.

**Methods:**

Data of patients with diabetes and HTN with regular follow-up visits in a multidisciplinary HTN treatment center from Brazil’s Midwest were retrospectively assessed. Patients ≥ 18 years enrolled in the service by June 2017 with a minimum of three visits were included. Anthropometric, blood pressure (BP), laboratory, pharmacological treatment, lifestyle, and cardiovascular events data were collected from first (V1), intermediate (V2) and most recent (V3) visits to the service. BP < 130 × 80 mmHg, LDL-cholesterol (LDL-C) < 70 mg/dL and HbA1C < 7.0% were defined as treatment targets. Wilcoxon signed-rank test was used to compare variables along study visits. A linear regression model was built to identify variables associated with better overall patient control.

**Results:**

A total of 162 patients were included (mean age of 56.5 ± 10.8 years). Median follow-up time was 60 (IQR 40–109) months, 80.2% of the sample was female and 83.3% had no cardiovascular event history. BP, total cholesterol, LDL-C, triglycerides and HbA1C values showed a significant trend to improve along the study visits (p < 0.001). Growing trend in aspirin (p = 0.045) and statins (p < 0.001) use was found, in addition to treatment compliance increase (p < 0.001). Significant improvement trends in BP (p < 0.001), LDL-C (p = 0.004) and HbA1C (p = 0.002) control were also found across visits. Control rates of BP, LDL-C and HbA1C in combination were low in V1, V2 and V3 (1.2, 1.9 and 6.8%, respectively), but showed significant improvement trend (p < 0.001). Treatment compliance (β-coefficient = 1.20; 95% CI 1.07–1.34; p < 0.001) was positively associated with better overall patients control.

**Conclusions:**

Multidisciplinary treatment of patients with diabetes and HTN significantly improved clinical and laboratory parameters, despite ageing of population evaluated. Although combined control of HbA1C, BP and LDL-cholesterol increased along follow-up, management of all these three conditions needs to improve, and focus on treatment compliance should be given to attain this goal.

## Background

Type 2 diabetes mellitus (T2DM) and hypertension (HTN) are multifaceted clinical syndromes commonly associated [1]. Since both are considered risk factors for coronary artery disease, cerebrovascular disease, renal failure and congestive heart failure, treatment of both conditions is essential [2]. Additionally, patients with diabetes are particularly vulnerable to hypertensive injury, and the coexistence of HTN has a significant impact on the poor prognosis of patients with diabetes because of its synergic deleterious effects on the micro and macro vasculature [3].

Since T2DM and HTN are degenerative diseases, their natural history is expected to be progressive, particularly when people adopt urban westernized lifestyles [4]. As a consequence both diseases tend to aggravate, and patients’ management become more challenging as times goes by [5]. Usually, in clinical practice, increasingly worse results in blood pressure (BP), hemoglobin A1C (HbA1C) levels and other risk factors control are found.

Multidisciplinary treatment for T2DM and HTN is recommended by most international guidelines addressing the two conditions individually [6–9], and much of these recommendations are due to the complexity of dealing with these patients. Although it is reasonable to assume that multidisciplinary treatment should be extended to patients with both T2DM and HTN, there is a lack of scientific literature supporting this hypothesis.

Aiming to report the results of a long-term multidisciplinary treatment approach for patients with HTN and T2DM treated in a Brazilian center, to show whether this treatment was capable to improve clinical and laboratory parameters, and additionally to identify strategies to improve the overall management of these patients we conducted this study.

## Methods

Data of patients with both HTN and T2DM with regular follow-up visits in a multidisciplinary HTN treatment center from Brazil’s Midwest were retrospectively assessed.

The center has been conducting its activities for more than 25 years, dedicated to hypertensive patients’ care, health professional’s teaching and research. The multidisciplinary team consists of physicians (general practitioners, cardiologists, endocrinologists and nephrologists), nurses, dietitians, physical therapists, physical education instructors, psychologists and musical therapists. Aiming to improve treatment compliance and reduce loss of follow-up, the maximum interval between each patient appointment is 3-months. Additionally, educational and health promotion activities are routinely performed with the patients [10]. Since the beginning of this multidisciplinary service, consultations have been registered in a standardized form. All healthcare professionals directly involved in patients care are routinely trained to fill this form, ensuring data reliability and reproducibility throughout the follow-up years [11].

This study included all patients with T2DM and HTN aged 18 years and older enrolled in the service by June 2017. A minimum of three visits to the service reported in the medical record was also required.

Hypertension was defined according to the 7th Brazilian Guideline of Arterial Hypertension (chapter 2): (1) office BP ≥ 140 × 90 mmHg; ambulatory BP monitoring (ABPM) ≥ 130 × 80 mmHg; (3) home BP monitoring ≥ 135 × ≥ 85 mmHg [12]. Patients receiving HTN treatment were also considered as hypertensives.

Diabetes definition followed the recommendations of the last Guidelines of the Brazilian Society of Diabetes [13]: (1) symptoms of polyuria, polydipsia and weight loss plus casual blood glucose ≥ 200 mg/dL (casual blood glucose—values obtained at any time of the day regardless of meal times); (2) fasting blood glucose ≥ 126 mg/dL (diagnosis should be confirmed by repeat testing on another day in case of small blood sugar elevations); (3) 2-h plasma glucose value after a 75-g oral glucose tolerance test ≥ 200 mg/dL. Diabetes treatment registered in medical records was also considered as diagnosis criteria.

### Data collection

Data from the first (V1), an intermediate (V2) and the most recent visit to the service (V3) were collected, regardless the healthcare professional responsible for the visit. V1 was defined as the first visit to the service after being diagnosed as diabetic. V2 was defined depending on the total number of visits (median point of the total number of visits, regardless the time elapsed between visits). V3 was defined as the most recent visit to the service. Follow-up time between visits was calculated from the differences between visits dates, and the results are given in months.

The following data were collected from medical records:

#### Anthropometric data

Weight, height and body mass index (BMI) calculated using the Quetelet formula (BMI = weight in kg/height^2^ in meter).

#### Blood pressure

A minimum of three BP measurements, with at least 1-min interval, was taken. All measurements were performed after 5 min of rest, on the upper limb, with the individual sitting and the arm supported. Appropriate cuffs were used depending on arm diameter. Mean values of last two measurements were considered for data analyses. BP measurements were performed with a mercury-column manometer until December 2012. From that date on, BP measurements were performed with oscillometric devices (OMRON semi-automatic equipment, model HEM-705CP). This routine was adopted in the service to avoid observer bias.

#### Laboratory data

- Renal function with creatinine measured in mg/dL;
- Glomerular filtration rate given in mL/min and estimated by the MDRD formula [14];
- Fasting glycemia and lipid profile: collected after a 12-h fasting, and observing the recommendation of no alcoholic beverage consumption in the preceding 48 h. The enzymatic colorimetric method was used to determine total cholesterol (TC), high density lipoprotein cholesterol (HDL), serum triglycerides (TG) and glycemia. The low density lipoprotein cholesterol (LDL) level was estimated with the Friedewald formula: LDL = TC − (HDL + TG/5) [15]. All values are given in mg/dL.

All tests were performed at the same laboratory, no more than 30 days prior to the visits.

#### Pharmacological treatment

- Anti-hypertensive drugs: assessing number of agents and use of angiotensin converter enzyme (ACE) inhibitors or angiotensin receptor blocker (ARB).
- Diabetes treatment: oral hypoglycemic agents and insulin.
- Other drugs: aspirin and statins.

Patients were also asked if they have been compliant to drug treatments since their most recent visit to the center (yes or no).

#### Lifestyle

- Smoking: current smoker or nonsmoker;
- Alcohol consumption: any alcohol consumption since the most recent visit;
- Sedentary lifestyle: no leisure physical activity, regardless duration and intensity.

#### Cardiovascular events

- Acute myocardial infarction (AMI): reported in medical records and confirmed by hospital discharge summary and/or high levels of myocardial necrosis biomarkers;
- Angina: reported in medical records and confirmed by hospital discharge summary;
- Stroke: reported in the medical record and confirmed by hospital discharge summary and/or imaging exam suggestive of cerebrovascular event;
- Coronary artery bypass grafting or angioplasty: reported in the medical record and confirmed by hospital discharge summary, surgeon’s report and/or angioplasty report.

### Cardiovascular risk estimation

The 10-year predicted cardiovascular risks were estimated by the Framingham risk function for total cardiovascular disease (CVD) risk [16]. The 10-year cardiovascular event rate for low, intermediate, high, and very high risk categories were respectively: < 10, 10–20, 20–30, and > 30%.

### BP, LDL-cholesterol and diabetes control definitions

Management of patients at this multidisciplinary center has always been based on national guidelines. The recommendations of these guidelines changed over time, leading to different treatment goals. Since, there was a great variation on visit dates even within V1, V2 and V3, and only minor changes in guidelines recommendations for diabetic patients occurred, we adopted the recommendations of the last Brazilian Guidelines of hypertension (chapter 8) [17], dyslipidemia [18] and diabetes [13] for the analyses.

- BP control was defined as systolic blood pressure (SBP) < 130 mmHg and diastolic blood pressure (DBP) < 80 mmHg.
- LDL control was defined as LDL < 70 mg/dL.
- HbA1C control as a proxy for diabetes control was defined as HbA1C < 7.0%.

### Multidisciplinary service set-up

The medical team assessed symptoms, lifestyle habits and medications being used, performed complete physical examination, interpreted complementary tests and established patient management (pharmacological and nonpharmacological treatments prescription; complementary tests request; and follow-up visits schedule). In addition, if acute clinical decompensation was identified in the medical visit, patients were referred to emergency care or hospitalization.

The nurse team assessed symptoms, vital signs, lifestyle habits and medications being used, in addition to instructing about treatment compliance in both pharmacological and nonpharmacological aspects. They defined the interval of the nurse follow-up appointment and referred patients for medical consultation if clinically necessary or to ensure a maximum interval between two medical visits of 6 months.

The group of dietitians emphasized nonpharmacological aspects of care, specifically the diet. They collected dietary data and assessed anthropometric data and vital signs. The management was aimed at dietary guidance with emphasis on salt restriction and prescription of diets for patients with diabetes.

The other health care professionals of the service did not conduct formal appointments, but rather a series of educational interventions to promote patients’ health. Physical therapists and physical education instructors conducted periodical meetings previously scheduled or met with patients at the waiting room. They emphasized the importance of regular physical activity; discussed preventive measures for injuries and falls; and additionally, they promoted assisted group physical activity for patients. Similarly, the psychology and musical therapy teams acted mainly in the waiting room, providing instructions and interventions aimed at stress reduction and waiting time improvement.

### Statistical analysis

Statistical analysis was performed using the software STATA V14 (StataCorp., College Station, Texas, USA). Continuous variables are presented as mean and standard deviation or median and interquartile range. Categorical variables are presented as *n* and %. Paired T-test was used to compare continuous variables and Chi Square test was used to compare categorical ones. The Wilcoxon signed-rank test was used to compare the variables along the study visits and results for the comparisons are presented as p value for trends [19]. A linear regression model was built to identify the variables independently associated to the number of conditions controlled (HTN, LDL-C and diabetes). Treatment compliance, age, follow-up time, sex, and number of anti-HTN were used as predictors in the model. The significance level adopted was p < 0.05.

## Results

A total of 162 patients were included, with a mean age of 56.5 ± 10.8 years. The median follow-up time was 60 (IQR 40–109) months, and the date of the first observation was May 20th, 1991. The majority of the sample was female (80.2%) and had no previous history of cardiovascular event (83.3%). The study population characteristics are presented in Table 1.

**Table 1:** Study population baseline characteristics

n	162
Age (years)	56.5 (± 10.8)
Female	130 (80.2%)
Body mass index (kg/m^2^)	30.9 (± 5.4)
Cardiovascular event^a^	27 (16.7%)
Predicted Framingham 10-year cardiovascular risk (%)	28.0 (± 18.0)
Follow-up time V1–V2 (months)^b^	34 (14–56)
Follow-up time V2–V3 (months)^b^	35 (19–53)
Total follow-up time (months)^b^	60 (40–109)

Values given in means (± SD) or n (%)

^a^Cardiovascular event—history of acute myocardial infarction, angina, cerebrovascular event, or revascularization (prior to study enrollment)

^b^Values give in median and interquartile range

BP, total cholesterol, LDL-cholesterol, triglycerides and HbA1C values showed a statistically significant trend to improve along the study visits. Oppositely, glomerular filtration rate values deteriorate throughout visits. Regarding lifestyle, alcohol consumption decreased across visits. Variables description along study visits are shown in Table 2.

**Table 2:** Variables description along study visits (n = 162), Goiânia—GO

	Visit 1	Visit 2	Visit 3	*p* value^a^
Systolic BP (mmHg)	140.2 (± 19.9)	136.2 (± 20.2)	133.5 (± 18.6)	0.002
Diastolic BP (mmHg)	87.7 (± 12.9)	83.7 (± 12.0)	79.6 (± 11.1)	< 0.001
Total cholesterol (mg/dL)	206.8 (± 52.0)	187.3 (± 47.1)	175.8 (± 43.2)	< 0.001
LDL-cholesterol (mg/dL)	120.8 (± 43.4)	107.6 (± 37.1)	100.3 (± 35.8)	< 0.001
HDL-cholesterol (mg/dL)	42.2 (± 9.9)	42.1 (± 10.3)	43.2 (± 9.9)	0.207
Triglycerides (mg/dL)	220.0 (± 160.7)	200.7 (± 173.7)	176.4 (± 125.7)	< 0.001
Fasting glucose (mg/dL)	149.6 (± 65.8)	137.0 (± 49.7)	147.8 (± 53.6)	0.515
HbA1C (%)	7.9 (± 1.6)	7.7 (± 1.7)	7.3 (± 1.5)	< 0.001
GFR (mL/min)	78.3 (± 21.5)	72.8 (± 21.1)	66.3 (± 21.0)	< 0.001
Smoke	8 (4.9%)	5 (3.1%)	5 (3.1%)	0.378
Alcohol consumption	14 (8.6%)	7 (4.3%)	5 (3.1%)	0.026
Sedentary lifestyle	20 (12.4%)	34 (21.0%)	26 (16.1%)	0.369
Predicted Framingham 10-year cardiovascular risk				
Low	19 (11.7%)	16 (9.9%)	15 (9.3%)	0.465
Intermediate	44 (27.2%)	53 (32.7%)	55 (33.9%)	0.188
High	42 (25.9%)	40 (24.7%)	40 (24.7%)	0.798
Very high	57 (35.2%)	53 (32.7%)	52 (32.1%)	0.556

Values given in means (± SD) or n (%)

*GFR* glomerular filtration rate

^a^*p* value for trends across visits; statistically significant at α < 0.05

Pharmacological treatment assessment showed a statistically significant growing trend in all variables along the study visits (number of anti-HTN, ACE inhibitors/ARBs, aspirin, statins, oral hypoglycemic agents, insulin, and treatment compliance) (Table 3).

**Table 3:** Medication variables description along study visits (n = 162), Goiânia—GO

	Visit 1	Visit 2	Visit 3	*p* value^a^
Number of anti-HTN	1.6 (± 0.9)	2.1 (± 0.9)	2.8 (± 0.8)	< 0.001
ACE inhibitors/ARBs	102 (63.0%)	134 (82.7%)	150 (92.6%)	< 0.001
Aspirin	27 (16.7%)	47 (29.0%)	52 (32.1%)	0.045
Statins	46 (28.4%)	66 (40.7%)	98 (60.5%)	< 0.001
Oral hypoglycemic agents	84 (51.9%)	146 (90.1%)	150 (92.6%)	< 0.001
Insulin	12 (7.4%)	19 (11.7%)	33 (20.4%)	0.001
Treatment compliance	55 (34.0%)	84 (51.9%)	111 (68.9%)	< 0.001

Values given in means (± SD) or n (%)

^a^*p* value for trends across visits; statistically significant at α < 0.05

**Fig. 1:**
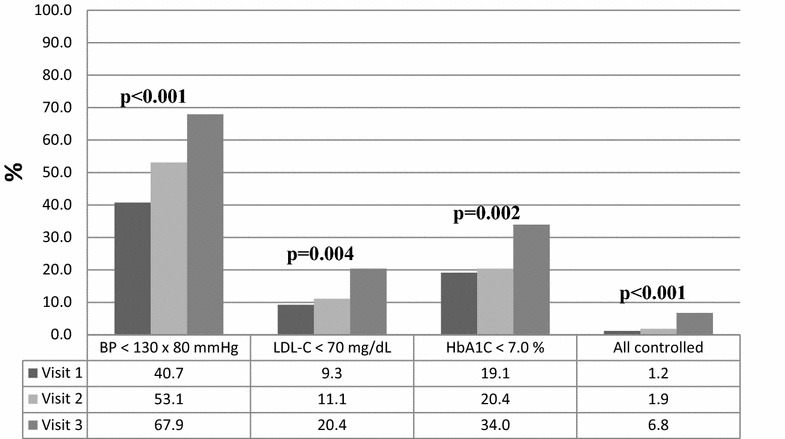
Blood pressure, LDL-cholesterol and HbA1C under control along study visits (n = 162). Goiânia—GO. *p* value for trends across visits; statistically significant at α < 0.05

Significant improvement trends in control of BP, LDL-cholesterol and diabetes were also found across study visits. When control of these three conditions was assessed in combination, low control percentages were found in V1, V2 and V3 (1.2, 1.9 and 6.8%, respectively), but also with a significant improvement trend (p < 0.001). These results are shown in Fig. 1.

A comparison between patients who achieved treatment goals in V3 to those who did not achieve was conducted to find which factors may have influenced the different results between groups. Treatment compliance was higher among patients with BP, LDL-cholesterol and diabetes controlled in V3, and was the only variable with significant difference in the groups’ comparison (Table 4).

**Table 4:** Patients who achieved treatment goals^a^ in visit 3 compared to those whom did not achieve (n = 162), Goiânia—GO

	Treatment goals in visit 3 Not achieved	Treatment goals in visit 3 Achieved	*p* value^b^
N	151	11	
Female	122 (80.8%)	8 (72.7%)	0.520
Age (years)	62.57 (± 11.54)	68.00 (± 10.15)	0.130
Total follow-up time (months)^c^	60 (37–105)	64 (51–150)	0.210
Number of anti-HTN	2.81 (± 0.84)	3.00 (± 0.77)	0.460
ACE inhibitors/ARBs	141 (93.4%)	9 (81.8%)	0.160
Body mass index (kg/m^2^)	30.77 (5.61)	27.48 (3.86)	0.057
Insulin	33 (21.9%)	0 (0.0%)	0.082
Oral hypoglycemic agents	141 (93.4%)	9 (81.8%)	0.160
Aspirin	47 (31.1%)	5 (45.5%)	0.330
Statins	94 (62.3%)	4 (36.4%)	0.090
Cardiovascular event^d^	48 (31.8%)	3 (27.3%)	0.760
Sedentary lifestyle	25 (16.6%)	1 (9.1%)	0.510
Smoke	5 (3.3%)	0 (0.0%)	0.540
Alcohol consumption	5 (3.3%)	0 (0.0%)	0.540
Treatment compliance	100 (66.7%)	11 (100.0%)	0.021
Predicted Framingham 10-year cardiovascular risk			
Low	14 (9.3%)	1 (9.1%)	0.980
Intermediate	50 (33.1%)	5 (45.4%)	0.400
High	38 (25.2%)	2 (18.2%)	0.600
Very high	49 (32.4%)	3 (27.3%)	0.720

Values given in means (± SD) or n (%)

^a^Blood pressure < 130 × 80 mmHg, LDL-cholesterol < 70 mg/dL, HbA1C < 7.0%

^b^Statistically significant at α = 0.05

^c^Values give in median and interquartile range

^d^Cardiovascular event—history of acute myocardial infarction, angina, cerebrovascular event, or revascularization

Aiming to identify ways to improve the overall control of the patient with diabetes and HTN we built a linear regression model assessing the variables independently associated with the number of conditions (BP, LDL-cholesterol and diabetes) under control in the most recent visit. In this analysis, treatment compliance (β-coefficient = 1.20; 95% CI 1.07–1.34; p < 0.001) was the only variable positively associated with a better overall control of these patients (Table 5).

**Table 5:** Linear regression coefficients for the number of diseases under control^a^ (n = 162), Goiânia—GO

Variables	*β*-coefficient	[95% CI]	*p* value^b^
Treatment compliant	1.20	1.07 to 1.34	< 0.001
Age	0.00	− 0.01 to 0.01	0.555
Follow-up time	0.01	− 0.02 to 0.03	0.869
Male sex	0.09	− 0.22 to 0.41	0.543
Number of anti-HTN	0.07	− 0.08 to 0.22	0.336

^a^Blood pressure < 130 × 80 mmHg, LDL-cholesterol < 70 mg/dL, HbA1C < 7.0%

^b^Statistically significant at α = 0.05

## Discussion

This is the first study showing the results of a multidisciplinary treatment strategy addressing specifically the patient with diabetes and hypertension. A significant trend to improve laboratorial and clinical parameters was found. Despite the low number of patients with BP, LDL and HbA1C controlled in combination along the study visits, a trend to improve this number was also found. Additionally, control rates of each condition individually improved along the study. The only variable independently associated with a better overall control of the patient with T2DM and HTN was treatment compliance.

Although BP, total cholesterol, LDL-cholesterol, triglycerides and HbA1C values showed a statistically significant trend to improve along the study visits, no changes in the predicted cardiovascular risk categories distribution were found. These results can be explained by the increasing age of the population along the study visits, since it is well stablished that age is a fundamental predictor of CVD risk [20].

A remarkable aspect of our results, which is clear from Fig. 1, is that blood pressure control was much better than the other factors. Although our multidisciplinary service has always focused on the overall patient management it is primarily specialized in hypertension treatment, which can explain this finding. Previous studies conducted in the same setting also showed good results in blood pressure management [11, 21] which must be simultaneously seen as extremely positive and as a call for action to improve management of other co-morbidities.

Morbidity in the diabetic patient is a consequence of both macro- and microvascular disease [22]. Once present, the progression of these complications can be slowed with drug interventions such as: ACE inhibitors or ARBs, aspirin, statins and optimization of oral hypoglycemic agents and insulin [23]. We found an increase in the number of individuals using ACE inhibitors/ARBs, aspirin, statins, oral hypoglycemic agents and insulin along the follow-up visits, suggesting an improvement in the pharmacological therapy prescribed. Similar results have been shown with the multidisciplinary treatment when addressing very elderly hypertensive patients [11].

The suboptimal management of patients with diabetes and HTN shown here by poor combined control of T2DM, HTN and dyslipidemia is consistent with previous reports from developed countries. The 1999–2000 US National Health and Nutrition Examination Survey (NHANES) found that only 7.3% of the adults with diabetes achieved the recommended therapeutic goals of HbA1c, BP and cholesterol [24], and this value improved, as shown along our follow-up, to 10.2% in the 2003–2006 NHANES survey [25]. Cross-sectional studies conducted in Poland [26] and China [27], less developed countries, found that among patients with diabetes and HTN, only 1.4 and 5.6% respectively, met all three targets. Even more concerning are the results from the randomized Steno-2 Study, conducted to evaluate the effect an intensified multifactorial intervention comprising behavior modification and polypharmacologic therapy aimed at several modifiable risk factors in patients with T2DM [28]. Although, the Steno-2 Study showed sustained beneficial effects with respect to vascular complications and on rates of cardiovascular deaths, it is notable that only one patient reached all five treatment goals (HbA1C, cholesterol, triglycerides, SBP and DBP) at the end of follow-up [28].

It is well established that HbA1C, BP and LDL-cholesterol control in each patient with diabetes and HTN needs to be optimized [29]. Therefore, the linear regression model we built assessing the variables independently associated with the number of conditions (BP, LDL-cholesterol and diabetes) under control, is a strength of our study that needs to be highlighted. In this model, treatment compliance was the only variable positively associated with management of patients with diabetes and HTN. Additionally to identifying treatment compliance as a variable associated with management of these patients, the simple and objective way we assessed compliance is another positive point. Instead of using complexes and sometimes unreliable methods to assess compliance [30], patients were asked if they have been compliant to drug treatments since their most recent visit to the center. This simple approach can be widely used in clinical practice and a negative answer to the question needs to warn and be a call for action for all health care providers dealing with these patients.

A potential limitation of this study is its design. We conducted a retrospectively single center with no control group study. Despite that, since all medical records are objective in this center, and the completion of its mandatory fields is exhaustively trained, the ability of generating reliable data is assured. Additionally, although a comparison with a control group would be ideal, the results we found can foster future studies and help informing the healthcare community about the optimal way to manage this specific population.

Another limitation is related to physical activity. Since we used just leisure physical activity in our definition, which accounts specifically for planned or formal physical activity (walking, running, cycling, swimming, strength training, etc.), daily physical activities were not considered. Therefore, the sedentary lifestyle results we found were probably overestimated.

Although more than 90% of patients with T2DM receive their routine care from primary care providers [31], a major unresolved controversy is the place of the generalist and the specialist in the treatment of these patients. Studies comparing care by specialists and generalists have generated conflicting findings [31–34]. In our case, care was delivered by a health care team in coordination with physicians from three different specialties: cardiology, nephrology and endocrinology. Notably, the presence of these three specialties is not required at the service concomitantly. Since we are reporting positive results the format adopted in our service can be a model for other centers handling patients diagnosed with both diabetes and HTN and aiming to implement a multidisciplinary strategy.

## Conclusion

Multidisciplinary treatment of patients with diabetes and hypertension has shown significant improvements in clinical and laboratory parameters, despite ageing of the population evaluated. Although combined control of HbA1C, BP and LDL-cholesterol increased along follow-up, management of all these three conditions needs to improve, and focus on treatment compliance should be given to attain this goal.

## Abbreviations

ABPM: ambulatory BP monitoring
ACE: angiotensin converter enzyme
AMI: acute myocardial infarction
ARB: inhibitors or angiotensin receptor blocker
BMI: body mass index
BP: blood pressure
CVD: cardiovascular disease
DBP: diastolic blood pressure
T2DM: type 2 diabetes mellitus
HbA1C: hemoglobin A1C
HDL: high density lipoprotein cholesterol
HTN: hypertension
LDL: low density lipoprotein cholesterol
SBP: systolic blood pressure
TC: total cholesterol
TG: serum triglycerides
V1: first visit
V2: intermediate visit
V3: most recent visit
